# Enhancement of CYP3A4 Activity in Hep G2 Cells by Lentiviral Transfection of Hepatocyte Nuclear Factor-1 Alpha

**DOI:** 10.1371/journal.pone.0094885

**Published:** 2014-04-14

**Authors:** Tsai-Shin Chiang, Kai-Chiang Yang, Ling-Ling Chiou, Guan-Tarn Huang, Hsuan-Shu Lee

**Affiliations:** 1 Institute of Biotechnology, National Taiwan University, Taipei, Taiwan; 2 School of Dentistry, College of Oral Medicine, Taipei Medical University, Taipei, Taiwan; 3 Liver Disease Prevention and Treatment Research Foundation, Taipei, Taiwan; 4 Department of Internal Medicine, National Taiwan University Hospital and National Taiwan University College of Medicine, Taipei, Taiwan; 5 Agricultural Biotechnology Research Center, Academia Sinica, Taipei, Taiwan; Montana State University, United States of America

## Abstract

Human hepatoma cell lines are commonly used as alternatives to primary hepatocytes for the study of drug metabolism *in vitro*. However, the phase I cytochrome P450 (CYP) enzyme activities in these cell lines occur at a much lower level than their corresponding activities in primary hepatocytes, and thus these cell lines may not accurately predict drug metabolism. In the present study, we selected hepatocyte nuclear factor-1 alpha (HNF1α) from six transcriptional regulators for lentiviral transfection into Hep G2 cells to optimally increase their expression of the CYP3A4 enzyme, which is the major CYP enzyme in the human body. We subsequently found that HNF1α-transfected Hep G2 enhanced the CYP3A4 expression in a time- and dose-dependent manner and the activity was noted to increase with time and peaked 7 days. With a multiplicity of infection (MOI) of 100, CYP3A4 expression increased 19-fold and enzyme activity more than doubled at day 7. With higher MOI (1,000 to 3,000), the activity increased 8- to 10-fold; however, it was noted the higher MOI, the higher cell death rate and lower cell survival. Furthermore, the CYP3A4 activity in the HNF1α-transfected cells could be induced by CYP3A4-specific inducer, rifampicin, and metabolized nifedipine in a dose-dependent manner. With an MOI of 3,000, nifedipine-metabolizing activity was 6-fold of control and as high as 66% of primary hepatocytes. In conclusion, forceful delivery of selected transcriptional regulators into human hepatoma cells might be a valuable method to enhance the CYP activity for a more accurate determination of drug metabolism *in vitro*.

## Introduction

Hepatocytes are the main cell type in human body and play a major role in the metabolism of drugs through the activity of their abundant cytochrome P450 (CYPs) enzymes. They consequently present themselves as the most reliable model cell in which to study drug metabolism *in vitro* [1], [2]. However, primary human hepatocytes are difficult to obtain and their quality can be variable. Although hepatocytes from animals, *e.g*., rodents, are commonly used in study of the drug metabolism, the drug metabolism properties of these hepatocytes do not always accurately reflect the metabolism processes that occur in human hepatocytes due to species difference [3]. Thus, studies based on animal-derived hepatocytes may not adequately predict drug metabolism in human hepatocytes [4], [5]. As such, human hepatoma cell lines have attracted significant research attention as convenient and reliable alternatives to primary human hepatocytes. In addition, these cell types can be easily obtained and maintained *in vitro*. However, their CYP enzyme levels are much lower than primary human hepatocytes [6]–[9], which may result in the inaccurate detection of certain drug metabolism processes [10].

There are 57 isoforms of CYP in the human liver. [2]. Among the CYP isoforms, CYP3A4 is known to play the key role in drug metabolism and it is responsible for the metabolism of more than half of all clinically used drugs. [11]–[14]. It is consequently an enzyme of interest for the study of drug metabolism processes in humans, and is particularly relevant to clinical and drug discovery research.

Many strategies have been used to augment CYP expression in human hepatoma cell lines. For example, the three-dimensional culture of human hepatoma (Hep G2) cells resulted in the mildly increased mRNA expression of CYP1A1, 1A2, 2B6, 2D6, and 3A4 [15]–[17]. In addition, the transfection of Hep G2 cells with recombinant adenoviruses that encode CYP 1A2, CYP2C9, and CYP3A4 has been shown to increase the activity of these enzymes remarkably to levels comparable to those detected in primary human hepatocytes [18]. However, additional studies regarding the use of viral transfection and the resulting effect on CYP expression are needed.

To further our understanding of CYP enzymes and to advance the use of hepatoma cells as replacements for primary hepatocytes, we investigated the expression and enzyme activity of CYP3A4 in Hep G2 cells following the delivery of hepatocyte nuclear factor-1 alpha (*HNF1α*) using a lentivirus system. HNF1α is a liver enriched transcription factor that has been shown to transactivate the promoters of several CYP genes *in vitro*, including CYP3A4 [13]. Following our protocol, the CYP3A4 enzyme activity of HNF1α-transfected cells increased significantly and transfected cells exhibited normal metabolic activity, as evidenced by their metabolism of a calcium channel blocker, nifedipine. Furthermore, the enzyme activity of transfected cells could be induced by a CYP3A4 inducer rifampicin.

## Materials and Methods

### Cell culture

Human embryonic kidney 293T (HEK293T) and human hepatoma Hep G2 cell lines were purchased from American Type Culture Collection (ATCC, Manassas, VA, USA). Cells were cultured in high glucose-Dulbecco's modified Eagle's medium (HG-DMEM, Gibco-BRL, Scotland, UK) supplemented with 10% fetal bovine serum (FBS) and 1× penicillin/streptomycin. Human hepatocytes were purchased from ScienCell Research Laboratories (Carlsbad, CA, USA). The hepatocytes were plated on flasks coated with poly-L-lysine (ScienCell Research Laboratories) and cultured in commercially available hepatocyte medium (ScienCell Research Laboratories). All the cells were incubated at 37°C under an atmosphere containing 5% CO_2_.

### Plasmid construction and recombinant lentivirus production

Plasmid construction and the production of recombinant lentiviruses doubly expressing reporter copGFP and hepatocyte nuclear factor (*HNF*)-*1α* or (HNF)-*1β*, sex determining region Y-box 17 (*SOX17*), X-box-binding protein 1 (*XBP-1*), pregnane X receptor (*PXR*), or retinoid X receptor (*RXR*) were obtained as described previously [19]. In brief, the cDNA was subcloned into pCDH cDNA Expression Lentivector (System Biosciences, Mountain View, CA, USA) under control of a CMV promoter. A separate promoter EF1 would constitutively promote the expression of the reporter copGFP. To produce recombinant lentiviruses, HEK293T cells were co-transfected with respective recombinant expression lentivectors in combination with envelop plasmid pMD2.G and packaging plasmid psPAX2 (deposited to Addgene, Cambridge, MA, by Dr. Didier Trono, School of Life Sciences and Frontiers in Genetics Program, Lausanne, Switzerland) using jetPEI transfection reagent (Polyplus-Transfection Inc., New York, NY, USA). The control virus was produced using the cloning vector without insertion.

### Titration of lentiviruses by flow cytometry

To determine the transfection unit of the obtained viruses, HEK293T cells were infected with a series of diluted virus solutions for 48 h prior to determination of the percentage of copGFP-positive cells by flow cytometry using a Becton Dickinson FACSCalibur flow cytometry system (BD Biosciences, Mississauga, Canada). WinMDI software was used for data analysis.

### Transduction of transcriptional regulators into Hep G2 cells by lentivirus infection

Hep G2 cells were seeded into 24-well plates at a density of 1×10^4^ cells/well in 0.5 mL culture medium. The cells were cultured for 1 day before being infected by indicated combinations and multiplicity of infection (MOI) of control or recombinant lentiviruses. The virus-infected Hep G2 cells were cultured for indicated days in 24-well plates and the medium was exchanged every other day.

### Total RNA isolation, reverse transcription, and quantitative-polymerase chain reaction (Q-PCR)

The total RNA of the cells was extracted using REzol™ C & T reagent (Protech Technologies, Taipei, Taiwan). Reverse transcription was performed with SuperScript III reverse transcriptase (Invitrogen, Carlsbad, CA, USA) with a total volume of 20 µL. For Q-PCR, a TaqMan system (Applied Biosystems, Foster City, CA, USA) with human *CYP3A4* primers/probes (Hs00604506_m1) was used with reference to glyceraldehyde-3-phosphate dehydrogenase (*GAPDH*, Hs02758991_g1) as a house-keeping gene. The Q-PCR conditions were as follows: denaturation at 95°C for 30 s, annealing at 60°C for 30 s, and extending at 72°C for 30 s for up to 40 cycles.

### Cell-based assays of various CYP activities

Cell-based assays were used to determine the cellular activities of CYP3A4, CYP2C9, and CYP1A1/1B1 (Promega Corp., Madison, WI, USA) according to manufacturer's instructions. Cells were incubated with respective luciferin substrates (Luciferin-IPA for CYP3A4, V9002; Luciferin-H for CYP2C9, V8791; and Luciferin-CEE for CYP1A1/1B1, V8751) for 1, 4, and 3 h, respectively, at 37°C with occasional mixing by swirling. Luciferin-IPA has been shown to be a sensitive and specific substrate for assaying CYP3A4 activity and the reaction is linearly correlated with recombinant CYP3A4 concentrations. It only minimally cross-reacts with the closely related enzymes CYP3A5 and 3A7 [20]. After incubation, an aliquot of 50 µL medium was transferred from each well to a 96-well opaque white luminometer plate at room temperature. Then, luciferin detection reagent (50 µL) was added to each well and the plate was allowed to stand for 20 min in the dark to initiate a luminescent reaction. The resulting luminescence was read using a Victor3 luminometer (PerkinElmer, Singapore). Following luminescence determinations, the genomic DNA was extracted from the cells (Geneaid Biotech, Cleveland, OH, USA) and the luminescence data were normalized to the respective amounts of genomic DNA to represent various CYP activities.

### Induction of CYP3A4 activity by rifampicin

Hep G2 cells were seeded into 24-well plates at a density of 1×10^4^ cells/well in normal culture medium. After 5 days at 37°C, 5% CO2, rifampicin (25 µM in dimethyl sulfoxide, DMSO) or the vehicle only (DMSO in the absence of rifampicin) was added into the culture medium. Cells were harvested following treatment for 48 h to determine the CYP3A4 activity. The induction fold is given by the mean CYP3A4 activity with rifampicin divided by the mean CYP3A4 activity with the vehicle only.

### Total protein extraction and western blot assay

Hep G2 cells infected with an HNF1α-expressing lentivirus and untreated control cells were cultured under normal conditions for one week. Cells were then washed twice with ice-cold phosphate-buffered saline (PBS). They were lysed in RIPA buffer [150 mM NaCl, 1 mM ethylenediaminetetraacetic acid, 1% Nonidet P-40, 1% sodium deoxycholate, 0.1% sodium dodecyl sulfate (SDS), 20 mM 3-(*N*-morpholino)propanesulfonic acid (MOPS), 1 mM phenylmethylsulfonyl fluoride, pH 7.0] on ice for 20 min. The lysates were centrifuged at 12,000×*g* for 5 min at 4°C and the supernatants were collected for analysis of the protein concentration using a Bio-Rad protein assay kit (Bio-Rad, Hercules, CA, USA). The proteins in the lysates (each 20 µg) were separated by 10% SDS- polyacrylamide gel electrophoresis (PAGE) and transferred to a Hybond™-C Extra membrane (Amersham Biosciences, Bath, UK). The transferred membrane was blocked in PBST buffer (0.1% Tween 20 in PBS) containing 5% skimmed milk powder for 1 h at room temperature. The membrane was then incubated in PBST containing a 1∶500 dilution of monoclonal anti-CYP3A4 primary antibody (Santa Cruz Biotechnology, Santa Cruz, CA, USA) overnight at 4°C. The membrane was subsequently washed three times in PBST, and then incubated in PBST containing goat anti-mouse secondary antibody (1∶5,000) conjugated with horseradish peroxidase (Jackson ImmunoResearch Laboratories, Inc., PA, USA). After washing, the membrane was developed using enhanced chemiluminescence (Millipore, Billerica, MA, USA). Blotting of β-actin was used as an internal control of the protein loading.

### Determination of cell survival and apoptosis after lentivirus infection at various MOI

Cell viability was determined by trypan blue staining to measure the rate of cell survival. The rate of apoptosis was determined by cell cycle analysis. Before the cell cycle analysis, cells in suspension were fixed in ice-cold 70% ethanol overnight, washed twice in ice-cold PBS, and finally resuspended in cold PBS. They were then stained *via* the addition of 20 µg mL^−1^ propidium iodide (Sigma-Aldrich, St. Louis, MO, USA), 0.1% Triton X-100, and 0.2 mg mL^−1^ DNase-free RNase A (Sigma-Aldrich) for 30 min at room temperature. Cells were analyzed by flow cytometry using a Becton Dickinson FACSCalibur flow cytometry system (BD Biosciences) and analyzed by WinMDI software.

### Nifedipine metabolism activity assay

To determine the cellular metabolic activity of CYP3A4, which is known to metabolize nifedipine (Sigma-Aldrich) into oxidized nifedipine (Sigma-Aldrich), cells were incubated in culture medium containing 10 µg mL^−1^ nifedipine for 24 h. The levels of oxidized nifedipine in the media of both untreated control and HNF1α-transduced Hep G2 cells were detected by high-performance liquid chromatography-electrospray tandem mass spectrometry, as described previously [19], and normalized to the respective amount of genomic DNA to give an indication of the nifedipine metabolism activity.

### Statistical analysis

Data are presented as mean ± SE. A one-way analysis of variance ANOVA with a post-hoc Dunnett's multiple comparison test was used to analyze the differences between the subgroups. For all statistical analyses, data from triplicates or three independent experiments were used. The statistical significance was set at *P*<0.05.

## Results

### Screening of regulators able to induce optimal CYP3A4 expression in Hep G2 cells

**Figure 1:**
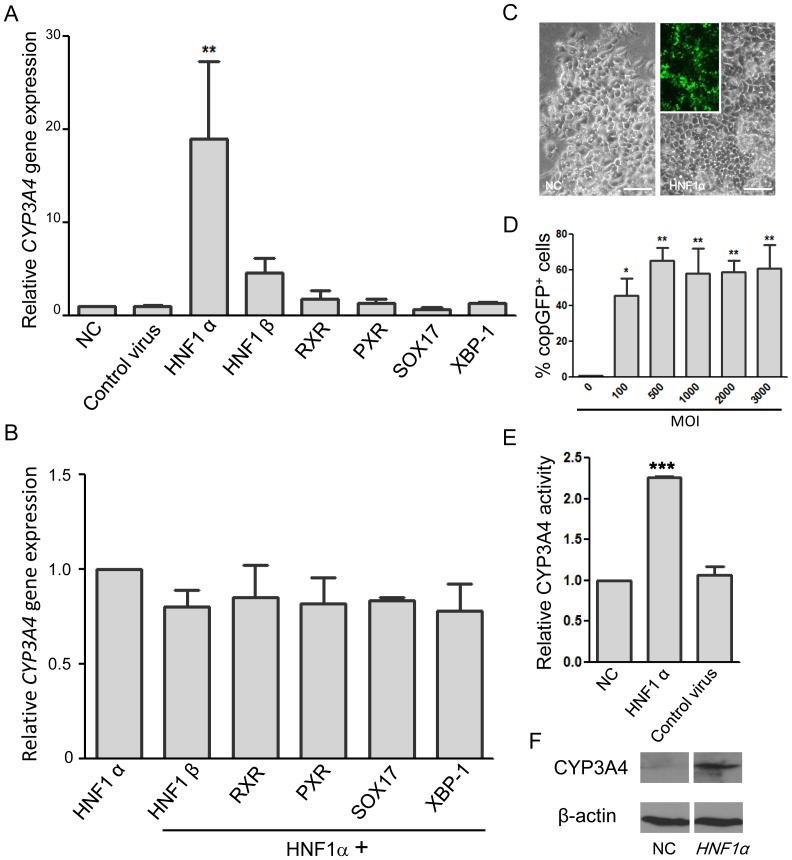
Selection of HNF1α as the optimal regulator for induction of CYP3A4 expression in Hep G2 cells. Cells were infected by control or indicated recombinant lentiviruses and cultured for 7 days before determination of the CYP3A4 expression and activity. (A) *CYP3A4* mRNA expression in normal control (NC) Hep G2 cells and cells infected (at MOI = 100) with control virus or recombinant lentiviruses respectively expressing *HNF1α*, *HNF1β*, *RXR*, *PXR*, *SOX17*, or *XBP-1*. Optimal *CYP3A4* expression occurs in cells infected with the HNF1α-expressing lentivirus. (B) Co-infection of Hep G2 cells with the HNF1α lentivirus and either HNF1β, RXR, PXR, SOX17, or XBP-1 did not increase *CYP3A4* mRNA expression levels. (C) Left panel: A representative bright field image of control HepG2 cells. Right panel: A representative bright field image and fluorescent image of HepG2 cells infected at MOI = 3,000 by lentiviruses containing *HNF1α*, showing the reporter copGFP fluorescence. Scale bar  = 100 µm. (D) Flow cytometry analysis showing percentage of copGFP^+^ Hep G2 cells 7 days post-infection with HNF1α-expressing lentivirus at various MOI. (E) Increased CYP3A4 enzyme activity and (F) protein levels in Hep G2 cells infected with the HNF1α-expressing lentivirus (MOI  = 100) compared with normal control cells (NC) or cells infected with control virus. **P<0.01, ***P<0.001 compared with NC cells.

Recombinant lentiviruses respectively expressing HNF1α, HNF1β, SOX17, XBP-1, PXR, and RXR (each at 100 MOI) were used to infect Hep G2 cells for 7 days to enhance their expression of *CYP3A4*. Among the six individually transduced transcriptional regulators, HNF1α significantly increase the expression of *CYP3A4* in Hep G2 cells 19-fold compared to control cells. Control virus did not affect the CYP3A4 expression and HNF1β, RXR, PXR, and XBP-1 have only very mild effects (Fig. 1A). Addition of another regulator to HNF1α did not result in a further increase of expression (Fig. 1B). In light of these findings, we selected HNF1α for further studies.

### HNF1α-enhanced CYP3A4 enzyme activity and protein levels in Hep G2 cells

Compared to control Hep G2 cells, HNF1α-transduced Hep G2 cells even at MOI = 3,000 did not show significant changes with regard to their morphology and displayed, as expected, green fluorescence associated with the copGFP reporter (Fig. 1C). In this study, transfection unit of the produced lentivirus and hence the MOI was determined on HEK293T cells. The infectivity of HNF1α-expressing lentivirus on Hep G2 cells was determined 7 days post-infection by flow cytometry to measure the rate of copGFP^+^ cells. The results showed the infection rates to be around 40% to 65% at MOI of 100 to 3,000 (Fig. 1D). After Hep G2 cells were transduced with HNF1α for 7 days, the enzymatic activity of CYP3A4 dramatically increased more than two-fold (Fig. 1E) and their CYP3A4 protein expression levels were also significantly increased (Fig. 1F).

### Effects of MOI and days post-HNF1α transduction on CYP3A4 activity in Hep G2 cells

**Figure 2:**
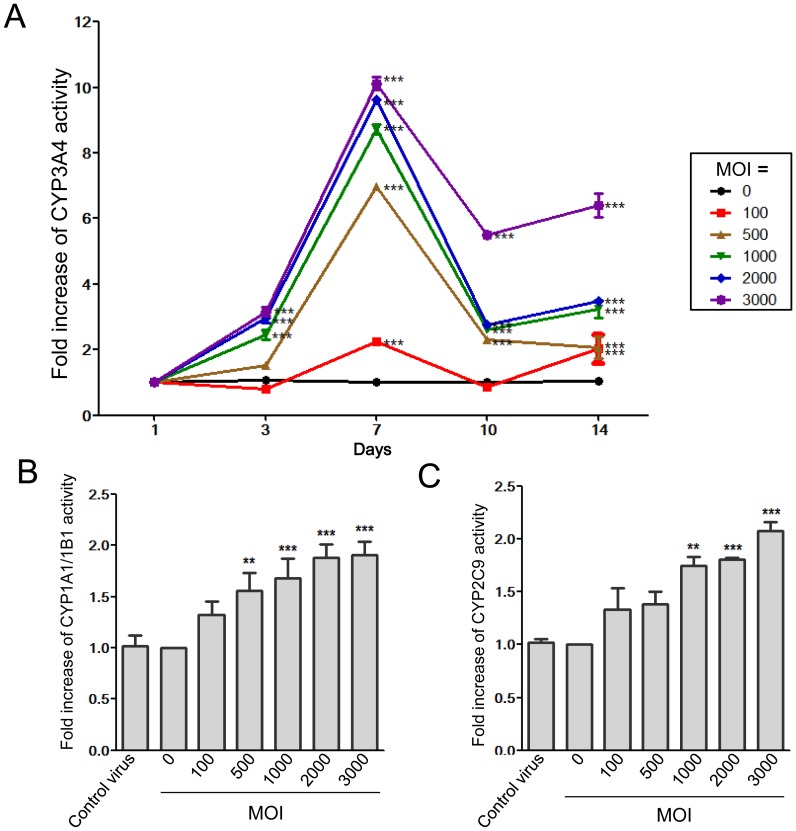
Enzyme activity of CYP3A4, CYP1A1/1B1, and CYP2C9 in HNF1α-transfected Hep G2 cells. (A) CYP3A4 activities in cells from day 1 to day 14 with various MOI are presented as the detected luminescence level normalized to the respective amount of genomic DNA. With various MOI, the activities peaked at day 7. With MOI≧500, the activities increased to 6–10 folds. At day 7, the activities of CYP1A1/1B1 (B) and CYP2C9 (C) dose-dependently increased to only around 2 folds at higher MOI. Control virus did not change the activities significantly. ***P<0.001 compared with normal control cells (MOI = 0).

In the absence of HNF1α transduction (MOI  = 0), the CYP3A4 enzymatic activity was steadily low from 0 to 14 days. At MOI ≧100, the CYP3A4 activity generally increased from day 3 to day 7, and then decreased thereafter to day 14. Furthermore, the MOI (100–3,000) was found to dose-dependently increase CYP3A4 activity (Fig. 2A). Given that the CYP3A4 activity peaks at day 7 after HNF1α transduction, the subsequent analyses were performed at that time-point. At MOI≧500, the increasing folds of CYP3A4 activity at day 7 were between 6 to 10 (Fig. 2A). In contrast, the increasing folds of CYP1A1/1B1 (Fig. 2B) and CYP2C9 (Fig. 2C) were only around 2 folds though there were dose-dependent effects. Control virus with MOI = 3,000 did not affect the activities of CYP1A1/1B1 and CYP 2C9 compared with normal control cells (MOI = 0).

### Influence of MOI on cell survival and apoptosis

**Figure 3:**
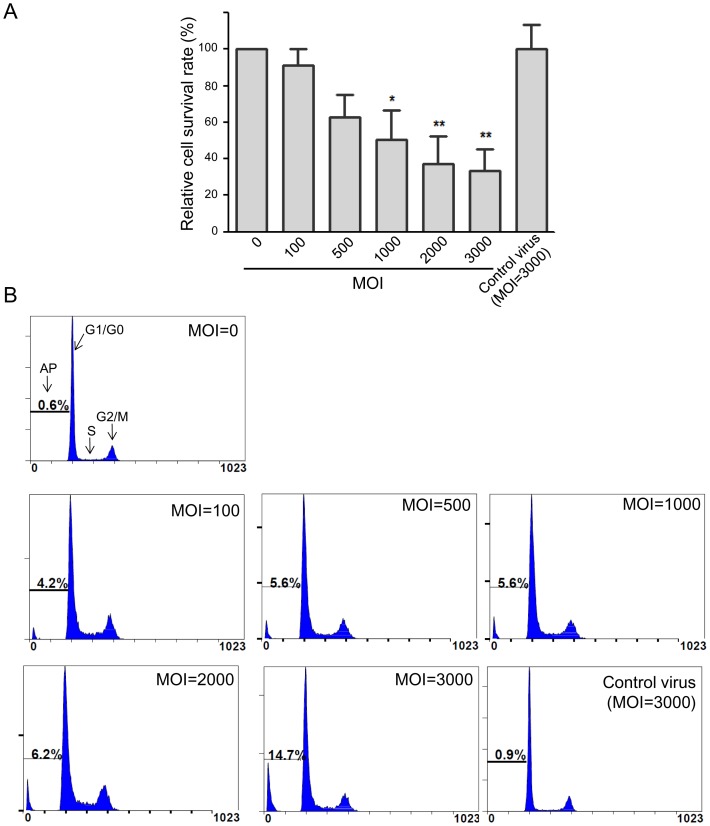
Characterization of HNF1α-transfected Hep G2 cells with various MOI 7 days post-infection. (A) The relative survival rate decreased as the viral MOI increased. (B) Cell cycle analysis revealed the percentage of apoptotic cells increased as the MOI increased. AP: apoptotic cells; G1/G0: G1/G0 phase; S: S phase; G2/M: G2/M phase. Control virus with MOI = 3,000 did not affect cell survival or apoptosis. *P<0.05, **P<0.01.

Cell survival and apoptosis were determined 7 days post-infection with the HNF1α-expressing lentivirus at various MOI to establish its effect on cell viability. The rate of cell survival decreased to below 40% as the MOI increased to 2,000. Control virus even at MOI = 3,000 did not affect cell survival (Fig. 3A).

Cell cycle analysis also confirmed that higher MOI result in an increased rate of apoptosis. In this regard, the apoptosis rate was found to be 0.6% at MOI  = 0 (control cells) and gradually increased to 4.2% at MOI  = 100, 5.6% at MOI  = 500 and MOI  = 1,000, 6.2% at MOI  = 2,000, and 14.7% at MOI  = 3,000 (Fig. 3B). Control virus even at MOI = 3,000 did not increase cell apoptosis rate compared with that in the control cells (0.9% *vs*. 0.6%), indicating that lentivirus infection per se was not the cause of cell death.

### Influence of HNF1α-transduction on the induction of CYP3A4 activity by rifampicin

**Figure 4:**
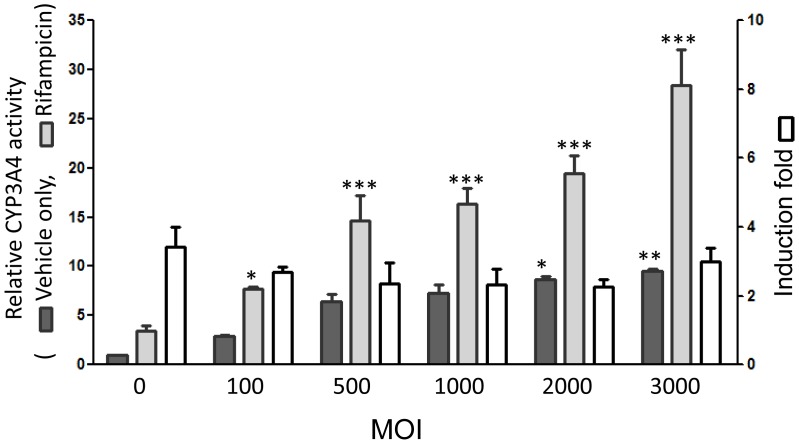
Inducibility of CYP3A4 enzyme activity by rifampicin in HNF1α-transfected Hep G2 cells at various MOI. Light gray bars: with rifampicin; dark gray bars: vehicle only; white bars: induction fold increase. *P<0.05, **P<0.01, ***P<0.001.

Rifampicin is known to induce CYP3A4 activity and, in the absence of HNF1α transduction (MOI  = 0), the induction fold increase in activity was 3.4. Importantly, the induction fold increase did not change as the MOI was varied and CYP3A4 activity was comparable (induction fold increases  = 2.7, 2.3, 2.2, 2.3, and 3.1 at MOI  = 100, 500, 1,000, 2,000, and 3,000, respectively; Fig. 4). This indicates that HNF1α-transduction of Hep G2 cells does not affect the induction of CYP3A4 activity by rifampicin.

### Nifedipine metabolism activity of HNF1α-transduced Hep G2 cells at day 7

**Figure 5:**
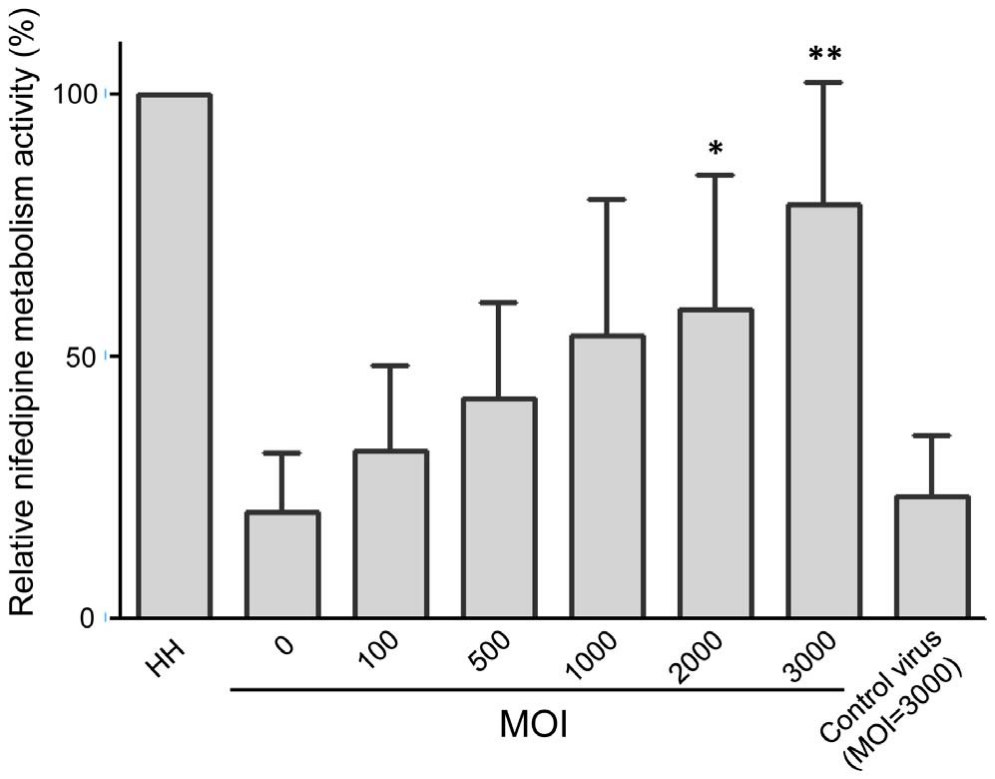
Nifedipine metabolism activity in HNF1α-transfected Hep G2 cells cultured for 7 days post-infection. The metabolic activity was determined by measuring the levels of oxidized nifedipine. Oxidized nifedipine was quantified by HPLC-ESI-MS/MS. HH: human hepatocytes used as positive control. The nifedipine metabolism activities increased as viral MOI increased. The activity of the cells infected by control virus with MOI = 3,000 is similar to that in normal control cells (MOI = 0). *P<0.05, **P<0.01.

Figure 5 shows the nifedipine metabolism activity of human hepatocytes and Hep G2 cells transduced by HNF1α at various MOI. In accordance with Figure 2 and Figure 4, these results show that higher MOI (100–3,000) result in an increased nifedipine metabolism activity in HNF1α-transduced Hep G2 cells at 7 days post-infection. The metabolism activity increased from 23.1% to 66.1% of the activity shown by human hepatocytes as the MOI was increased from 0 to 3,000. The metabolism activity in Hep G2 cells infected with control virus at MOI = 3,000 was at similar level as in control Hep G2 cells.

## Discussion

In this study, we selected HNF1α as a candidate transcriptional regulator to optimally increase the expression and activity of CYP3A4 in Hep G2 cells (Fig. 1). We found that it increased CYP3A4 activity in a dose-dependent manner (Fig. 2, 4, and 5) and infected cells still responded to rifampicin, a known CYP3A4 inducer (Fig. 4). However, heavier lentivirus infection resulted in an increased rate of cell death and diminished cell survival (Fig. 3).

Although human hepatoma cell lines are commonly used as alternatives to primary human hepatocytes in drug metabolism assays, their expression of CYP enzymes is usually too low to predict metabolism correctly [9]. In this regard, our study aimed to increase CYP3A4 expression in a commonly used human hepatoma cell line, Hep G2.

Several studies have revealed that transcriptional regulators, including transcription factors and nuclear receptors, are important in controlling *CYP3A4* gene expression [2], [5], [9], [21]–[23]. For example, HNF1 (composed of HNF1α and HNF1β hetero- or homo-dimers) is an important regulator that controls liver development and liver-specific expression of several *CYP* genes[24]–[26], while XBP-1 is an essential transcription factor also involved in liver development and the ER stress response [24], [25]. SOX17 is another critical regulator and is involved in the differentiation of stem cells into the hepatic lineage; it also contributes to the expression of *CYP* genes in differentiated hepatocyte-like cells [27].

On the other hand, PXR, which is an orphan nuclear receptor, is known to be the major regulator of the CYP3A subfamily in their response to the exposure of various xenobiotics [28], [29], while RXR is thought to form a heterodimer with PXR to activate the expression of CYP3A4 [5].

In the present study, we transfected Hep G2 cells with lentiviruses respectively expressing HNF1α, HNF1β, SOX17, XBP-1, PXR, and RXR to elevate *CYP3A4* expression. To our surprise, transfection of PXR and RXR showed only a minimal effect on *CYP3A4* expression in Hep G2 cells. In contrast, HNF1α transfection markedly increased expression, as shown in Figure 1A. Interestingly, addition of an additional regulator (HNF1β, SOX17, XBP-1, PXR, or RXR) to HNF1α did not enhance *CYP3A4* expression (Fig. 1B). Elevation of CYP3A4 functional activity and protein levels by HNF1α transfection was confirmed by a functional assay and western blotting (Fig. 1E and F). Previous works using a spheroid culture of Hep G2 cells for 12 days showed only around a 50% increase of *CYP3A4* mRNA expression compared to control cells [15]–[17]. In contrast, HNF1α-transfected Hep G2 cells in this study markedly increased the expression up to nineteen times compared to control cells. Although direct transfection of *CYP* genes into cells can increase CYP3A4 enzyme activity remarkably to levels comparable to human hepatocytes, such highly over-expressed enzyme levels may preclude the detection of possible drug-drug interactions that are mediated by acting on the same nuclear receptors [15], [18], [30]–[32]. Induction of CYP3A4 activity with rifampicin in our study showed that it is still possible to detect drug-drug interactions in treated cells, while increasing the expression of CYP3A4. Moreover, the HNF1α-transduction of Hep G2 cells did not affect the induction fold increase of CYP3A4 activity following treatment with rifampicin regardless of the MOI (Fig. 4).

HNF1α transduction of CYP3A4 activity was found to be time-dependent and peaked at 7 days post-infection, after which point, the activity declined (Fig. 2). This decrease in activity may result from either the gradual loss of the *HNF1α* transgene from the cells or a possible growth advantage of non-virus infected cells in the culture. The increased activity of CYP3A4 was dose-dependent with regard to lentivirus infection and reached a plateau of an eight- to ten-fold increase with an MOI of 1,000.

Rifampicin is a ligand inducer able to bind and activate PXR to induce CYP3A4 activity. Hep G2 cells are known to express PCR-detectable levels of PXR constitutively. Therefore, the activity of CYP3A4 in both control and HNF1α-transfected Hep G2 cells can be induced by treatment with rifampicin (Fig. 4). Interestingly, the forced expression of PXR in Hep G2 cells did not markedly increase CYP3A4 expression (Fig. 1A). Epigenetic regulation may represent the possible reason why the forced expression of PXR does not significantly increase CYP3A4 expression because the PXR binding site on the CYP3A4 gene promoter in the human liver is highly methylated [9], [33]. To assess the potential of using HNF1α-transduced Hep G2 cells in drug metabolism prediction, we assessed the metabolism of nifedipine in transduced cells [34]–[36]. Our results demonstrate that the transduced Hep G2 cells exhibited drug metabolism activities at a level of around 70% of primary human hepatocytes (Fig. 5).

In conclusion, transduction of certain transcriptional regulators in hepatoma cells may be a useful strategy to establish an assay system to more accurately study drug metabolism *in vitro*.

## References

[1] Meyer UA (1996) Overview of enzymes of drug metabolism. J Pharmacokinet Biopharm 24: 449–459. 9131484 10.1007/BF02353473

[2] Xu C, Li CY, Kong AN (2005) Induction of phase I, II and III drug metabolism/transport by xenobiotics. Arch Pharm Res 28: 249–268. 15832810 10.1007/BF02977789

[3] Pritchard JF, Jurima-Romet M, Reimer ML, Mortimer E, Rolfe B, et al (2003) Making better drugs: Decision gates in non-clinical drug development. Nat Rev Drug Discov 2: 542–553. 12815380 10.1038/nrd1131

[4] Jones SA, Moore LB, Shenk JL, Wisely GB, Hamilton GA, et al (2000) The pregnane X receptor: a promiscuous xenobiotic receptor that has diverged during evolution. Mol Endocrinol 14: 27–39. 10628745 10.1210/mend.14.1.0409

[5] Wang H, LeCluyse EL (2003) Role of orphan nuclear receptors in the regulation of drug-metabolising enzymes. Clin Pharmacokinet 42: 1331–1357. 14674787 10.2165/00003088-200342150-00003

[6] Hart SN, Li Y, Nakamoto K, Subileau EA, Steen D, et al (2010) A comparison of whole genome gene expression profiles of HepaRG cells and HepG2 cells to primary human hepatocytes and human liver tissues. Drug Metab Dispos 38: 988–994. 20228232 10.1124/dmd.109.031831 PMC2879958

[7] Antherieu S, Chesne C, Li R, Camus S, Lahoz A, et al (2010) Stable expression, activity, and inducibility of cytochromes P450 in differentiated HepaRG cells. Drug Metab Dispos 38: 516–525. 20019244 10.1124/dmd.109.030197

[8] Donato MT, Lahoz A, Castell JV, Gomez-Lechon MJ (2008) Cell lines: a tool for in vitro drug metabolism studies. Curr Drug Metab 9: 1–11. 18220566 10.2174/138920008783331086

[9] Castell JV, Jover R, Martinez-Jimenez CP, Gomez-Lechon MJ (2006) Hepatocyte cell lines: their use, scope and limitations in drug metabolism studies. Expert Opin Drug Metab Toxicol 2: 183–212. 16866607 10.1517/17425255.2.2.183

[10] Huang YS, Chern HD, Su WJ, Wu JC, Chang SC, et al (2003) Cytochrome P450 2E1 genotype and the susceptibility to antituberculosis drug-induced hepatitis. Hepatology 37: 924–930. 12668988 10.1053/jhep.2003.50144

[11] Guengerich FP (2008) Cytochrome p450 and chemical toxicology. Chem Res Toxicol 21: 70–83. 18052394 10.1021/tx700079z

[12] Luo G, Guenthner T, Gan LS, Humphreys WG (2004) CYP3A4 induction by xenobiotics: biochemistry, experimental methods and impact on drug discovery and development. Curr Drug Metab 5: 483–505. 15578943 10.2174/1389200043335397

[13] Martinez-Jimenez CP, Jover R, Donato MT, Castell JV, Gomez-Lechon MJ (2007) Transcriptional regulation and expression of CYP3A4 in hepatocytes. Curr Drug Metab 8: 185–194. 17305497 10.2174/138920007779815986

[14] Tarantino G, Di Minno MN, Capone D (2009) Drug-induced liver injury: is it somehow foreseeable? World J Gastroenterol 15: 2817–2833. 19533803 10.3748/wjg.15.2817 PMC2698999

[15] Gunness P, Mueller D, Shevchenko V, Heinzle E, Ingelman-Sundberg M, et al (2013) 3D organotypic cultures of human HepaRG cells: a tool for in vitro toxicity studies. Toxicol Sci 133: 67–78. 23377618 10.1093/toxsci/kft021

[16] Nakamura K, Kato N, Aizawa K, Mizutani R, Yamauchi J, et al (2011) Expression of albumin and cytochrome P450 enzymes in HepG2 cells cultured with a nanotechnology-based culture plate with microfabricated scaffold. J Toxicol Sci 36: 625–633. 22008537 10.2131/jts.36.625

[17] Leite SB, Wilk-Zasadna I, Zaldivar JM, Airola E, Reis-Fernandes MA, et al (2012) Three-dimensional HepaRG model as an attractive tool for toxicity testing. Toxicol Sci 130: 106–116. 22843569 10.1093/toxsci/kfs232

[18] Tolosa L, Donato MT, Perez-Cataldo G, Castell JV, Gomez-Lechon MJ (2012) Upgrading cytochrome P450 activity in HepG2 cells co-transfected with adenoviral vectors for drug hepatotoxicity assessment. Toxicol In Vitro 26: 1272–1277. 22138474 10.1016/j.tiv.2011.11.008

[19] Chiang TS, Yang KC, Zheng SK, Chiou LL, Hsu WM, et al (2012) The prediction of drug metabolism using scaffold-mediated enhancement of the induced cytochrome P450 activities in fibroblasts by hepatic transcriptional regulators. Biomaterials 33: 5187–5197. 22541353 10.1016/j.biomaterials.2012.04.014

[20] Cali JJ, Sobol M, Ma D, Uyeda HT, Meisenheimer P (2009) CYP3A4 P450-Glo Assays with luciferin-IPA: the most sensitive and selective bioluminescent CYP3A4 assay. Promega Corporation Web site. http://worldwide.promega.com/resources/pubhub/cellnotes/cyp3a4-p450-glo-assays-with-luciferin-ipa-the-most-sensitive-and-selective-bioluminescent-cyp3a4/

[21] Odom DT, Zizlsperger N, Gordon DB, Bell GW, Rinaldi NJ, et al (2004) Control of pancreas and liver gene expression by HNF transcription factors. Science 303: 1378–1381. 14988562 10.1126/science.1089769 PMC3012624

[22] Honkakoski P, Negishi M (2000) Regulation of cytochrome P450 (CYP) genes by nuclear receptors. Biochem J 347: 321–337. 10749660 10.1042/0264-6021:3470321 PMC1220963

[23] Jover R, Bort R, Gómez-Lechón MJ, Castell JV (1998) Re-expression of C/EBPα induces CYP2B6, CYP2C9 and CYP2D6 genes in HepG2 cells. FEBS Lett 431: 227–230. 9708908 10.1016/s0014-5793(98)00746-7

[24] Reimold AM, Etkin A, Clauss I, Perkins A, Friend DS, et al (2000) An essential role in liver development for transcription factor XBP-1. Genes Dev 14: 152–157. 10652269 PMC316338

[25] Leung T, Rajendran R, Singh S, Garva R, Krstic-Demonacos M, et al (2013) Cytochrome P450 2E1 (CYP2E1) regulates the response to oxidative stress and migration of breast cancer cells. Breast Cancer Res 15: R107. 24207099 10.1186/bcr3574 PMC3979157

[26] Akiyama TE, Gonzalez FJ (2003) Regulation of P450 genes by liver-enriched transcription factors and nuclear receptors. Biochim Biophys Acta 1619: 223–234. 12573481 10.1016/s0304-4165(02)00480-4

[27] Takayama K, Inamura M, Kawabata K, Tashiro K, Katayama K, et al (2011) Efficient and directive generation of two distinct endoderm lineages from human ESCs and iPSCs by differentiation stage-specific SOX17 transduction. PLoS One 6: e21780. 21760905 10.1371/journal.pone.0021780 PMC3131299

[28] Moore LB, Goodwin B, Jones SA, Wisely GB, Serabjit-Singh CJ, et al (2000) St. John's wort induces hepatic drug metabolism through activation of the pregnane X receptor. Proc Natl Acad Sci U S A 97: 7500–7502. 10852961 10.1073/pnas.130155097 PMC16574

[29] Moore JT, Kliewer SA (2000) Use of the nuclear receptor PXR to predict drug interactions. Toxicology 153: 1–10. 11090943 10.1016/s0300-483x(00)00300-0

[30] Elizondo G, Medina-Dıíaz IM (2003) Induction of CYP3A4 by 1α,25-dyhydroxyvitamin D3 in HepG2 cells. Life Sciences 73: 141–149. 12738030 10.1016/s0024-3205(03)00262-5

[31] Cooper BW, Cho TM, Thompson PM, Wallace AD (2008) Phthalate induction of CYP3A4 is dependent on glucocorticoid regulation of PXR expression. Toxicol Sci 103: 268–277. 18332045 10.1093/toxsci/kfn047

[32] Choi S, Sainz B Jr, Corcoran P, Uprichard S, Jeong H (2009) Characterization of increased drug metabolism activity in dimethyl sulfoxide (DMSO)-treated Huh7 hepatoma cells. Xenobiotica 39: 205–217. 19280519 10.1080/00498250802613620 PMC2953797

[33] Kacevska M, Ivanov M, Wyss A, Kasela S, Milani L, et al (2012) DNA methylation dynamics in the hepatic CYP3A4 gene promoter. Biochimie 94: 2338–2344. 22906825 10.1016/j.biochi.2012.07.013

[34] Wang XD, Li JL, Lu Y, Chen X, Huang M, et al (2007) Rapid and simultaneous determination of nifedipine and dehydronifedipine in human plasma by liquid chromatography-tandem mass spectrometry: Application to a clinical herb-drug interaction study. J Chromatogr B Analyt Technol Biomed Life Sci 852: 534–544. 10.1016/j.jchromb.2007.02.026 17339138

[35] Patki KC, Von Moltke LL, Greenblatt DJ (2003) In vitro metabolism of midazolam, triazolam, nifedipine, and testosterone by human liver microsomes and recombinant cytochromes p450: role of cyp3a4 and cyp3a5. Drug Metab Dispos 31: 938–944. 12814972 10.1124/dmd.31.7.938

[36] Haas DM, Quinney SK, McCormick CL, Jones DR, Renbarger JL (2012) A pilot study of the impact of genotype on nifedipine pharmacokinetics when used as a tocolytic. J Matern Fetal Neonatal Med 25: 419–423. 21644845 10.3109/14767058.2011.583700

